# Ketamine-Based Anesthetic Protocols and Evoked Potential Monitoring: A Risk/Benefit Overview

**DOI:** 10.3389/fnins.2016.00037

**Published:** 2016-02-16

**Authors:** Nicoleta Stoicea, Gregory Versteeg, Diana Florescu, Nicholas Joseph, Juan Fiorda-Diaz, Víctor Navarrete, Sergio D. Bergese

**Affiliations:** ^1^Department of Anesthesiology, The Ohio State University Wexner Medical CenterColumbus, OH, USA; ^2^College of Medicine, The Ohio State University Wexner Medical CenterColumbus, OH, USA; ^3^Faculty of Medicine, Carol Davila University of Medicine and PharmacyBucharest, Romania; ^4^Department of Neuroscience, The Ohio State UniversityColumbus, OH, USA; ^5^Servicio de Anestesiologia, Clínica Cira GarcíaLa Habana, Cuba; ^6^Department of Neurosurgery, The Ohio State University Wexner Medical CenterColumbus, OH, USA

**Keywords:** ketamine, evoked potentials, motor evoked potentials, somatosensory evoked potentials, general anesthesia, drug abuse, phencyclidine

## Abstract

Since its discovery, ketamine, a non-competitive N-methyl D-aspartate (NMDA) receptor antagonist related to phencyclidine, has been linked to multiple adverse reactions sometimes described as “out of body” and “near death experiences,” including emergence phenomena, delusions, hallucinations, delirium, and confusion. Due to these effects, ketamine has been withdrawn from mainstream anesthetic use in adult patients. Evoked potentials (EPs) are utilized to monitor neural pathways during surgery, detect intraoperative stress or damage, detect and define the level of neural lesions, and define abnormalities. Unfortunately, many of the volatile anesthetics commonly used during spinal and neurologic procedures suppress EP amplitude and monitoring. Ketamine has been found in several preclinical and clinical studies to actually increase EP amplitude and thus has been used as an analgesic adjunct in procedures where EP monitoring is critical. Once the gap in our knowledge of ketamine's risks has been sufficiently addressed in animal models, informed clinical trials should be conducted in order to properly incorporate ketamine-based anesthetic regimens during EP-monitored neurosurgeries.

## Introduction

In 1977, Marcia Moore, a famous author of New Age books, shared her “cosmatrix” ketamine experiences with Dr. Alltounian, a clinical anesthesiologist who attended one of Moore's workshops. After only two ketamine “trips,” they became engaged. One year later, Moore disappeared on a cold January night after injecting herself with ketamine (Domino, 2010).

The ketamine journey began in the early 1950's when a group of scientists from Park Davis MI initiated studies leading to the synthesis of phencyclidine (PCP) on March 26, 1956. PCP was responsible for a centrally mediated sensory deprivation syndrome, concluded John Stirling Meyer, M.D., Head of Neurology at Wayne State University. Doctor Bratton, Head of Pharmaceutical Research at Park Davis, stated that a shorter-acting derivative of the drug would limit emergence delirium. One of the short-acting agents produced in his laboratory was known as “ketamine.” The first human recipient of a subanesthetic, intravenous (IV) dose of ketamine in August, 1964, described strange experiences “like floating in outer space.” After a long discussion, the researchers decided to call ketamine a “dissociative anesthetic,” because of its ability to disconnect human subjects from their environment. In 1973, the drug-scheduling system classified ketamine as a schedule III. Years later, ketamine was considered schedule II because of its increasing frequency of abuse (Domino, 2010).

Over time, ketamine was found to have multiple clinical applications in psychiatry and anesthesiology. It has been reported that ketamine is effective during the acute treatment of Major Depressive Disorder episodes with suicidal ideation. Moreover, recent studies concluded that a single sub-anesthetic dose of ketamine was capable of decreasing depression scores in patients with major or bipolar depression (Zarate et al., 2006; Permoda-Osip et al., 2015). In 2011, Shlamovitz et al. reported a case of severe respiratory failure in a patient with a history of asthmatic exacerbations that benefited from a dissociative dose of ketamine to avoid intubation when standard therapies failed (Shlamovitz and Hawthorne, 2011). Ketamine has long been used as an analgesic with multiple routes of administration, including oral, IV, transdermal, and intranasal, to help relieve post-operative pain (Azevedo et al., 2000). In addition, ketamine can be used as an anesthetic during electroconvulsive therapy when a patient cannot tolerate other anesthetics (Rasmussen et al., 1996).

However, due to its sympathomimetic effect, ketamine use in anesthetic procedures complicates the monitoring of anesthesia depth, the level of hypnosis, and presents a more difficult titration process to a proper anesthetic dose. It was also reported to increase the difficulty of performing the wake-up test and delayed the moment of extubation (Gruber and Morley, 1999; Frei et al., 2007). Airway monitoring is also mandatory during ketamine-induced anesthesia due to its effects on glandular secretions, triggering respiratory blockage and laryngospasm even if airway reflexes are maintained (Stevenson, 2005).

As an abused drug, ketamine is acquired under various “street names” including “Special K.” As a recreational “club drug,” ketamine is used worldwide because of its affordability and dissociative and hallucinatory effects (Lim et al., 2008; Corazza et al., 2013). In 2014, Goyal et al. published a case report of an anesthesiologist abusing ketamine and benzodiazepines in an attempt to cope with a stressful environment (Goyal et al., 2014).

Since its discovery, ketamine has been linked to multiple adverse reactions including emergence phenomena, delusions, hallucinations, delirium, and confusion, sometimes described as “out of body” and “near death experiences” (Treston et al., 2009).

## The dissociative effects of ketamine: pharmacological bases

Ketamine is a non-competitive N-methyl D-aspartate (NMDA) receptor antagonist that exerts its effect on cortical and spinal neurons. (Martin and Lodge, 1985, 1988) Ketamine has a pKa of 7.5, a molecular weight of 238 Da, and high neuronal membrane permeability suggesting the intracellular action of the drug (Reich and Silvay, 1989; Lester et al., 2015). Chemically, ketamine is closely related to PCP (Reich and Silvay, 1989). In addition to its antagonistic effects on NMDA receptors, ketamine has been shown to disrupt dopaminergic neurotransmission and impair cognitive function associated with the prefrontal region of the brain. This in turn triggers the release of glutamate with an excitatory effect on post-synaptic, non-NMDA glutamate receptors (Moghaddam et al., 1997).

The effects of ketamine are wide-reaching and affect sobriety and pain perception, sight and hearing, somatosensory perception and short-term memory, as well as vigilance and disposition. Both the (S) and (R) enantiomers of ketamine induce these effects at differing intensities (Oye et al., 1992).

Francois et al. suggested that acute changes to event-related O_2_ signaling in the anterior cingulate cortex may be responsible for the slowed reaction times observed in ketamine users (Francois et al., 2015).

Behrens et al. reported that repeated preclinical exposure to ketamine (30 mg/kg × 2) is responsible for an increase in brain superoxide, with a disinhibition of glutaminergic activity and increased excitatory transmission in the prefrontal cortex. This excitotoxicity may explain the observed symptoms and impaired function shared by chronic and subchronic ketamine users, as well as schizophrenic patients (Behrens et al., 2007; Sorce et al., 2010; Schiavone et al., 2013). Ketamine is believed to enact these alternations through initial activation of proinflammatory cytokine, interleukin-6 (IL-6), which in turn activated the superoxide-producing enzyme, nicotinamide adenine dinucleotide phosphate oxidase-2 (NOX2; Behrens et al., 2008; Stone et al., 2014).

## Current clinical applications

The prolonged periods of sedation observed after ketamine-based anesthetic regimens in children and the increased rate of emergence delirium have incited further controversy. However, a recent study conducted by Treston et al. in pediatric patients (*n* = 745) receiving IV ketamine reported that only 2.1% of patients developed emergence delirium. Moreover, ketamine-induced hallucinations appeared to be equally pleasant for children as terrifying for adults (Treston et al., 2009).

Newer studies have also revealed that the short-term memory loss produced by ketamine administration is not actually caused by a disturbance of memory, but as a result of general sensory perception impairment (Oye et al., 1992). Due to these effects, ketamine was withdrawn from mainstream anesthetic use in adult patients (Morgan and Curran, 2012). The profound alterations made by ketamine use to a person's cognitive state along with emerging research reports that chronic ketamine abuse is linked to thalamic alterations and psychotic symptoms similar to the changes seen in schizophrenia, have provoked research into ketamine's effects on the brain (Stone et al., 2014).

One study found that ketamine was responsible for neurodegenerative processes in the rat brain when infused continuously for 9 h in developing mice. Braun et al. demonstrated that ketamine toxicity is dose dependent, triggering apoptosis at low concentrations and necrosis at higher concentrations (Morgan and Curran, 2012).

Recently, we noted a trend of increasing interest in analyzing the effects of ketamine on evoked potential (EP) monitoring during spinal and neurological surgeries under general anesthesia.

### Ketamine and evoked potentials

EPs are utilized to monitor neural pathways during surgery, detect intraoperative stress or damage, detect neural lesions, define the level of lesions, and define abnormalities. Visual, brainstem auditory, somatosensory evoked potentials (SSEPs), and motor evoked potentials (MEPs) are all widely known and used in clinical practice (Nuwer, 1998; Braun et al., 2010). Unfortunately, many commonly employed anesthetic agents suppress EP amplitudes, greatly limiting the anesthesiologist during EP-monitored surgeries.

Furmaga et al. (2014) analyzed the effects of ketamine and propofol on MEPs in rodents with and without a conditioning deep brain stimulus (DBS). The same lab had previously shown that DBS increases the amplitude of MEPs. Three groups of rodents were assigned to receive the following treatments: the first group received a loading dose of ketamine, 75 mg/kg intramuscularly, followed by IV infusion at 125 μg/kg/min, the second and third group received propofol, 10 mg/kg IV, followed by IV infusion at either 400 μg/kg/min for the standard dose group and 800 μg/kg/min for the high dose group. It was found that the anesthetics had a statistically significant effect on MEP. Without DBS, propofol infusion progressively suppressed the MEP amplitude while under ketamine anesthesia the amplitude did not change over time. The amplitude of MEP under ketamine anesthesia was greater than the amplitude measured under the highest dose of propofol but there was no statistically significant difference between the propofol groups. With different frequencies of DBS applied, under ketamine an increase in the DBS pulse frequency resulted in a progressive increase in MEP amplitude, reaching statistical significance at 50 and 100 Hz, but no such effect was observed under the different rates of propofol infusion. The results showed that ketamine was capable of increasing the amplitude of MEPs with and without a conditioning stimulus (Mason, 2004).

Independently, Iyer et al. (2010) performed an analysis of MEP recording on rodents before and after a moderate spinal cord injury. One group of rodents was given isoflurane anesthesia and the other group was given ketamine, xylazine, and atropine. Measurements of MEPs showed an almost complete suppression of MEPs in the isoflurane group while the ketamine group showed an ability to maintain the amplitude of these potentials (Furmaga et al., 2014).

Publications espousing the benefits of ketamine-based anesthetic regimens for EP-monitored procedures have not been limited to rodents. In 2005, Erb et al. reported the case of a 4 year old child scheduled to undergo an expansion thoracoplasty for congenital scoliosis, with no other known abnormalities or comorbidities. Anesthesia was induced with sevoflurane, 50% nitrous oxide, and 50 μg of fentanyl. Sevoflurane was stopped after induction and propofol (50–100 μg/kg/min) and remifentanil (2 μg/kg/min) started. During this surgery, no MEPs could be recorded in the tibialis anterior muscle but post-operative motor function was normal. Four months after the original surgery the patient underwent another planned procedure that required monitoring of MEPs. The same monitoring procedures were applied; anesthesia was induced with a 20 mg bolus of ketamine, followed by an infusion of ketamine (4 mg/kg/h) and remifentanil (2 μg/kg/min). During this surgery, MEP responses were obtained in the upper and lower extremities throughout the procedure, and spatial facilitation was applied for the lower extremities. The report emphasized ketamine's ability to maintain MEP recording in a patient where propofol previously inhibited MEP monitoring. Study investigators concluded that EP monitoring in children <8 y.o. of height 70–180 cm, whose small stature seems to have an inverse correlation with the threshold stimulus intensity necessary for MEP recording, was impossible during propofol/N_2_O anesthesia. When ketamine was added to this anesthesia regimen, MEP monitoring was possible in these young subjects. Importantly, propofol/N_2_O anesthesia did not inhibit MEP monitoring in patients over the age of eight. Thus, patient age is an important factor to consider in planning the anesthetic regimen for EP-monitored surgeries, and ketamine may have particular use in the MEP monitoring of very young patients (Iyer et al., 2010).

In 1994, Kalkman et al. randomized five healthy subjects in three separate sessions, to receive either pentobarbital (1.5 mg/kg), droperidol (0.07 mg/kg), or ketamine (1.00 mg/kg), IV. The purpose of this study was to determine if these agents could be used to reduce the likelihood of patient awareness during surgery while still allowing MEP monitoring. A minimum interval of 1 week between drug administrations with subsequent evaluation was allowed to ensure no overlap of drug effects. After receiving the IV drug, subjects were stimulated using transcranial magnetic stimulation. MEPs in the contralateral adductor pollicis were recorded at 2, 4, 6, 10, 15, 20, and 30 min following injection. Droperidol depressed the amplitude of both MEP recordings but onset latency was unchanged. Pentobarbital produced variable changes in the amplitude; some MEPs were unchanged, and some were substantially depressed, though no significant amplitude change was observed overall. Ketamine increased the MEPs 150–220% in three of the five subjects within 10 min of injection. Though clearly underpowered, the study concluded that droperidol can create sustained amplitude depression, pentobarbital had a variable effect, producing substantial MEP depression in certain subjects, and ketamine increased amplitude in some patients while leaving it unchanged in others. This study concluded that ketamine was able to “facilitate elicitation” of evoked myogenic responses in the tibialis anterior, an increase in amplitude of 150–250% being noted. The authors encouraged further assessment of the interactions between ketamine and opioids/nitrous oxide in humans, as a potential anesthetic combination, able to ease intraoperative monitoring of MEP (Erb et al., 2005).

Langeron et al. (1997) compared ketamine-midazolam and fentanyl-midazolam anesthesia with 60% nitrous oxide in oxygen in subjects undergoing spinal fusion surgery under general anesthesia. Either ketamine (3 mg/kg) or fentanyl (6 μg/kg) was given IV during induction. Anesthesia was then maintained through an infusion of 2 mg/kg/h ketamine or 3 μg/kg/h fentanyl. The results were recorded while the subject was awake, at the end of induction, at 15 min, 1, and 2 h after induction, during skin closure, and after discontinuation of nitrous oxide. A significant decrease in cortical somatosensory evoked potential (CSSEP) amplitude between the pre-induction value and other time-point values was observed in the fentanyl-midazolam group. The ketamine-midazolam group did not display any statistically significant changes in the CSEP recordings between time points. No significant difference in CSSEP amplitude was noted between the fentanyl-midazolam and ketamine-midazolam groups when compared at a single time point. The authors concluded that, at the present, there appears to be no advantage to using ketamine during spine surgery requiring CSSEP monitoring. This study analyzed data obtained from 20 subjects with 10 subjects per group, reducing the statistical power of the results. Furthermore, the use of 60% of N_2_O is likely to have affected the results, due to its confounding depressant effect on CSSEPs. The study concluded that ketamine demonstrated no benefit during major spinal surgery when CSSEP monitoring is required (Kalkman et al., 1994).

Schubert et al. (1990) studied ketamine's effect on EPs before and after administration of 70% N_2_O, a concentration known to be MEP depressant. Ketamine was given IV as a 2 mg/kg bolus and then maintained by continuous infusion at a rate of 30 μg/kg/h. SSEPs from median nerve stimulation were monitored at various time points (before induction, post-induction, after N_2_O was introduced, and after N_2_O was discontinued). The results indicated that ketamine significantly enhanced the cortical amplitudes of SSEPs prior to N_2_O administration. However, when N_2_O was added, ketamine was unable to prevent the depressant action on SSEP amplitudes, though the authors did stated that MEP amplitudes appeared higher than expected when only N_2_O was administered. Of note, 6 (38%) out of the 16 study subjects required labetalol during the study (Langeron et al., 1997).

Furthermore, Ubags et al. (1997) performed a study on surgical patients to determine the effect of ketamine and etomidate on MEPs from myogenic transcranial stimulation when supplementing sufentanil/N_2_O anesthesia. Anesthesia was induced with etomidate (0.3 mg/kg) and sufentanil (1.5 mg/kg) and subsequently maintained with sufentanil (0.5 mg/kg/h) and 50% N_2_O. Before any surgical interventions that might have damaged spinal cord functioning and at least 60 min after induction of anesthesia, either a bolus of 0.5 mg/kg ketamine or a bolus of etomidate 0.1 mg/kg was administered. Transcranial MEPs to stimulation were recorded before and after drug administration. No significant change in either amplitude or latency after ketamine administration was observed. Etomidate resulted in an amplitude reduction during the first 5 min after administration; however no significant changes in amplitude were noted after 5, 10, and 15 min. This study concluded that both ketamine and etomidate can be administered during MEP monitoring, though transient reductions in amplitude should be anticipated when etomidate is used (Schubert et al., 1990).

Another case involving the use of ketamine to counter the depressive effects of N_2_O and sevoflurane was reported by Penney (2010). The case report described a 15 year old girl weighing 48 kg who underwent a 9 h scoliosis correction surgery with SSEP and MEP monitoring. The anesthesia was induced with 6% sevoflurane and 60% nitrous oxide. Fentanyl (250 μg), lorazepam (1 mg), and rocuronium (30 mg) were also administered intravenously. Sevoflurane was eventually discontinued when IV anesthesia was started with 0.4–0.6 mg/kg/h of ketamine, 0.9–1.2 μg/kg/h dexmedetomidine, and 1–2 μg/kg/h of fentanyl. The 60% nitrous oxide in 100% oxygen was continued during the entire length of the procedure. Fentanyl was discontinued 30 min prior to the wake-up test being performed, and dexmedetomidine and ketamine were stopped 5–10 min before test administration. Following successful test completion, the patient then received a 50 mg bolus of propofol, a 1 mg bolus of lorazepam, and new infusions of 1.2 μg/kg/h dexmedetomidine, 0.5 μg/kg/h ketamine, and 1 μg/kg/h fentanyl. During surgery, successful intraoperative SSEP and MEP monitoring was performed and the patient exhibited no neurological or motor deficits after surgery (Ubags et al., 1997).

A similar report by Agarwal et al. (2008) described a 12 year old female scheduled to undergo an anterior/posterior fusion of her lumbar spine. The patient had a surgical history of a spinal astrocytoma resection with a partial thoracic laminectomy 3 years prior. A thoracic spinal fusion had also been performed 1 year prior. Leg weakness was noted upon preoperative evaluation. IV ketamine (175 mg), midazolam (5 mg), and d-tubocurare (25 mg) were used to induce anesthesia. During the case, IV midazolam (0.1 mg/kg/h) and ketamine (0.8 mg/kg/h) were used for anesthesia maintenance without volatile agents. For the entire length of the procedure, SSEPs were able to be reliably monitored on the right side but not the left. Upon closure, 50 μg of fentanyl were administered, significantly decreasing SSEP amplitude and prolonging the EP latency (Penney, 2010).

In this patient's previous thoracic anterior/posterior spinal fusion, sufentanil-based anesthesia was used in addition to 70% nitrous oxide and vecuronium. Although identical techniques and equipment for SSEP monitoring were used, no adequate EPs could be obtained, providing support for the superiority of ketamine-midazolam-based anesthesia regimens during SSEP monitoring for spinal surgery (Penney, 2010).

A recent article proposed the use of propofol-based total intravenous anesthesia (TIVA) and opioid-based anesthesia for optimal MEP monitoring. The article advised avoidance of volatile anesthetics, as these agents suppress potentials (Agarwal et al., 1998). Different studies displayed the advantages of ketamine over isoflurane, pentobarbital, and droperidol when motor and sensory EPs are monitored (Iyer et al., 2010).

## Conclusion and future directives

Regardless of its benefits, ketamine is no longer favored as a mainstream anesthetic, primarily because of its ability to trigger emergence delirium, delusions, hallucinations, and confusion, leading to a prolonged period of post-operative sedation and recovery. Due to the promising effects of ketamine-use as an adjuvant anesthetic during EP-monitored neurosurgeries, it is worthwhile to further investigate ketamine's anti-inflammatory and anti-excitotoxic, dose-dependent effects in preclinical studies. However, the clinical use of ketamine should always be based upon the risk/benefit evaluation. Once the gap in our knowledge of ketamine's risks has been sufficiently addressed in animal models, informed clinical trials should be conducted in order to properly incorporate ketamine-based anesthetic regimens during EP-monitored neurosurgeries.

## Author contributions

All authors listed, have made substantial, direct, and intellectual contribution to the work, and approved it for publication.

### Conflict of interest statement

The authors declare that the research was conducted in the absence of any commercial or financial relationships that could be construed as a potential conflict of interest.

## Abbreviations

PCP: Phencyclidine
IV: Intravenous
NMDA: N-methyl D-aspartate
GABA: Gamma Aminobutyric Acid
PV: Parvalbumin
IL-6: Interleukin-6
NOX2: Nicotinamide Adenine Dinucleotide Phosphate Oxidase-2
ROS: Reactive Oxygen Species
EP: Evoked potential
SSEP: Somatosensory Evoked Potential
MEP: Motor Evoked Potentials
DBS: Deep Brain Stimulation
CSSEP: Cortical Somatosensory Evoked Potential
TIVA: Total Intravenous Anesthesia.
